# Investigation of the Microbiome of Industrial PDO Sfela Cheese and Its Artisanal Variants Using 16S rDNA Amplicon Sequencing and Shotgun Metagenomics

**DOI:** 10.3390/foods13071023

**Published:** 2024-03-27

**Authors:** Natalia Tsouggou, Aleksandra Slavko, Olympia Tsipidou, Anastasios Georgoulis, Svetoslav G. Dimov, Jia Yin, Constantinos E. Vorgias, John Kapolos, Marina Papadelli, Konstantinos Papadimitriou

**Affiliations:** 1 Department of Food Science and Technology, University of the Peloponnese, 24100 Kalamata, Greece; n.tsouggou@go.uop.gr (N.T.); a.slavko@go.uop.gr (A.S.); i.kapolos@uop.gr (J.K.); m.papadelli@uop.gr (M.P.); 2 Laboratory of Food Quality Control and Hygiene, Department of Food Science and Human Nutrition, 18855 Athens, Greece; olibiatsip@gmail.com; 3 Department of Biochemistry and Molecular Biology, National and Kapodistrian University of Athens, Panepistimioupolis-Zographou, 15784 Athens, Greece; tgeorgoulis@med.uoa.gr (A.G.); cvorgias@biol.uoa.gr (C.E.V.); 4 Faculty of Biology, Sofia University “St. Kliment Ohridski”, 8, Dragan Tzankov Blvd., 1164 Sofia, Bulgaria; svetoslav@biofac.uni-sofia.bg; 5 Hunan Provincial Key Laboratory of Animal Intestinal Function and Regulation, College of Life Sciences, Hunan Normal University, Changsha 410081, China; jiayin@hunnu.edu.cn; 6 Hunan International Joint Laboratory of Animal Intestinal Ecology and Health, College of Life Science, Hunan Normal University, Changsha 410081, China

**Keywords:** Sfela cheese, 16S rDNA, shotgun metagenomics, binning, lactic acid bacteria, microbiota

## Abstract

Sfela is a white brined Greek cheese of protected designation of origin (PDO) produced in the Peloponnese region from ovine, caprine milk, or a mixture of the two. Despite the PDO status of Sfela, very few studies have addressed its properties, including its microbiology. For this reason, we decided to investigate the microbiome of two PDO industrial Sfela cheese samples along with two non-PDO variants, namely Sfela touloumotiri and Xerosfeli. Matrix-assisted laser desorption/ionization–time of flight mass spectrometry (MALDI-TOF MS), 16S rDNA amplicon sequencing and shotgun metagenomics analysis were used to identify the microbiome of these traditional cheeses. Cultured-based analysis showed that the most frequent species that could be isolated from Sfela cheese were *Enterococcus faecium*, *Lactiplantibacillus plantarum*, *Levilactobacillus brevis*, *Pediococcus pentosaceus* and *Streptococcus thermophilus*. Shotgun analysis suggested that in industrial Sfela 1, *Str. thermophilus* dominated, while industrial Sfela 2 contained high levels of *Lactococcus lactis.* The two artisanal samples, Sfela touloumotiri and Xerosfeli, were dominated by *Tetragenococcus halophilus* and *Str. thermophilus*, respectively. *Debaryomyces hansenii* was the only yeast species with abundance > 1% present exclusively in the Sfela touloumotiri sample. Identifying additional yeast species in the shotgun data was challenging, possibly due to their low abundance. Sfela cheese appears to contain a rather complex microbial ecosystem and thus needs to be further studied and understood. This might be crucial for improving and standardizing both its production and safety measures.

## 1. Introduction

White brined cheeses constitute an essential category of cheese produced worldwide and they are an important part of the diet of Mediterranean and Balkan regions. The white brined cheeses, also known as “pickled cheeses”, owe their name to the distinctive white color and the high salt brines used during their manufacturing [1,2]. These cheeses lack rinds and vary in texture and moisture levels, ranging from soft to semi-hard, with a pleasantly acidic and salty taste that becomes piquant as they ripen. Their unique characteristics are mainly attributed to the lactic acid fermentation process taking place in the initial steps of production, as well as during ripening in brine, which may last for several months. These traits have been developed over centuries of household or artisanal practices. 

Sfela cheese has been recognized as PDO (protected designation of origin) according to the Official Government Gazette of the Hellenic Parliament (25/18.01.94). It is a Greek white brined cheese produced in the Messinia and Laconia regional units, located in the southern Peloponnese. According to the PDO regulation, milk used for Sfela production can be ovine, caprine, or a mixture of both. Traditional rennet or enzymes can be added, while in the case of pasteurized milk, cultures of lactic acid bacteria (LAB) can be used. The coagulation of the milk is conducted at 30–32 °C, and the resulting curd is cut into small pieces and cooked at a temperature between 38°C and 40 °C with continuous stirring. The curd is transferred to cheesecloth to drain the whey and then placed on a cheese rack where it is slightly pressed. Subsequently, the curd is sliced into strips called “sfelides”, dry salting is applied, and it is moved into barrels or metal containers filled with a 20 Bé brine. Finally, the cheese is left at room temperature for one month before being transferred to cold rooms (4–6 °C), where it ripens for at least three months. When kept sealed under brine, the cheese can be stored for up to two years [3]. The desired characteristics of Sfela are a maximum moisture content of 45% and a minimum fat-in-dry matter of 40%. Based on studies, the chemical composition of the cheese is estimated to be approximately 28% fat, 21% protein, 6% salt and 12% salt in moisture [4]. Sfela is known for its distinctively spicy and salty taste. Interestingly, it has been suggested that when it comes to marketing, this cheese has limited export potential [5].

Today, manufacturing of white brined cheeses may include the addition of starter cultures. Several starters have been evaluated, including *Streptococcus thermophilus*, *Lactobacillus delbrueckii* ssp. *bulgaricus* and *Lactococcus lactis* [6,7]. Mixtures of mesophilic and thermophilic LAB strains are also frequently employed as they have the ability to cause fast acidification of the milk, which is necessary for cheese manufacturing. The selection of the starter cultures is an essential step in the production of white brined cheeses using pasteurized milk to ensure safety while maintaining consistent quality and sensory properties. Besides that, adjunct cultures have been suggested as potential additives to further enhance the flavor, functional attributes, or safety properties of white brined cheeses. Overall, adjunct cultures may offer the potential to improve cheese quality and even create a probiotic dynamic [8,9,10,11]. Additional members of the microbiome of white brined cheeses may include various species and may be shifted throughout the ripening process. For instance, *Enterococcus faecalis*, *Enterococcus durans*, *Pediococcus pentosaceus* and *Pediococcus acidilactici* were initially present in high numbers in fresh Feta cheese but declined during ripening and were outgrown by lactobacilli [12]. Counts for lactobacilli may increase during the first stage of ripening. Lactobacilli are favored by low pH and high salt content, with *Lactiplantibacillus plantarum* found to be the predominant species in Feta cheese [13]. These bacteria are non-starter LAB (NSLAB) and can be detected in raw milk, enduring the initial heat treatment, or can be transferred to the cheese and brine from the surrounding environment [14]. Species of *Leuconostoc* (*Leuconostoc lactis* and *Leuconostoc mesenteroides* subsp. *dextranicum*) have also been found in Beyaz Peynir cheese. Coliforms can also be present in brined cheeses, but their numbers have been found to decrease during the reduction of pH [15]. Micrococci can contribute to the development of flavor and aroma. Still, their numbers are usually low in white brined cheeses due to the inhibitory effect of the low pH of the cheese and brine [16]. The microbiome of white brined cheeses may additionally contain various yeasts. Species including *Saccharomyces cerevisiae*, *Debaryomyces hansenii*, *Candida famata*, *Pichia membranifaciens*, *Torulaspora delbrueckii*, *Kluyveromyces marxianus*, *Candida sake*, and *Kluyveromyces lactis* have been previously found in Feta cheese [17,18,19]. In Domiati cheese, some of the yeast species that have been previously identified are *Issatchenkia orientalis*, *Candida albicans*, *Clavispora lusitaniae* (*Candida lusitaniae*), *Kodamaea ohmeri* (*Pichia ohmeri*), *K. marxianus*, and *Candida catenulate* [20]. It is worth noting that, some yeast species, such as *C. albicans* and other common yeast pathogens, which are found in fresh cheese and brines, are probably unable to survive the maturation process [21]. An overgrowth of yeasts may cause defects to the white brined cheeses like early blowing, characterized by the presence of gas holes [22].

Furthermore, next generation sequencing (NGS) approaches are gaining momentum as they can provide a more precise identification of rare or difficult-to-culture microbial populations. Metagenomics, a culture-independent approach, is widely used for studying microbial ecosystems in various foods, including traditional dairy and fermented products [23]. Currently, two main tools are employed: amplicon sequencing for identifying the microbial populations mostly at the genus level and shotgun metagenomics analysis for insights into the composition and functional properties of species (or even subspecies) in a niche. Both techniques have been previously applied in studies conducted for the characterization of various white brined cheese microbiomes. More specifically, amplicon-based sequencing for the identification of both bacterial and yeasts genera has been employed in the case of various Turkish white cheeses including Tulum and Ezine [24,25,26], Greek artisanal cheeses like Kalathaki Limnou, Gidotyri and Batzos, [27,28,29], Brazilian Minas cheese [30] and the Cypriot cheeses Halloumi and Halitzia [31,32]. Feta, which is the most popular Greek cheese, has been selected as the basis for multiple studies employing amplicon sequencing and/or shotgun metagenomics to explore its microbiome under different technological conditions [33,34,35].

While several studies have been conducted on the microbiology of Greek white brined cheeses, to our knowledge, there is a notable gap in the study of Sfela cheese, despite its PDO status and its significance for the local producers in the Peloponnese. Therefore, in this study, we combined a culture-based approach with 16S rDNA amplicon-based sequencing and shotgun metagenomics to initiate a first investigation of the microbiome of Sfela cheese, which has not been reported in the literature before. Throughout this study, two different variants of Sfela were also investigated, namely Sfela touloumotiri and Xerosfeli, which were included to provide insight into these traditional cheeses’ microbial ecosystems. 

## 2. Materials and Methods

### 2.1. Cheese Sampling

In this study, a total of four cheese samples were selected for analysis, including two PDO industrial Sfela samples and two non-PDO artisanal variants (Xerosfeli and Sfela touloumotiri). All samples analyzed were from different producers. For industrial Sfela 1 and the two artisanal variants, a mixture of ovine and caprine milk was used, while industrial Sfela 2 was produced from ovine milk solely. In all cases, milk was pasteurized with the exception of Sfela touloumotiri that was thermised. Both the industrial PDO samples and the two Sfela variants were produced in locations that fulfilled the geographical requirements specified for PDO Sfela cheese. Furthermore, the industrial Sfela cheese samples had been ripened for at least three months, as stipulated by PDO regulations. Τhe non-PDO Xerosfeli was produced in a similar manner to PDO Sfela, but with a shorter ripening duration that may be distributed without being immersed in brine (“xero” means dry in Greek). The Sfela touloumotiri cheese was ripened in a touloum (animal skin bag) for an unknown period of time. All the samples were carefully transferred to the laboratory and stored at a temperature of 4 °C until analysis.

### 2.2. Growth Conditions and Strain Isolation

To initiate the isolations of the different Sfela strains, ten grams (10 g) of the cheese samples were homogenized in 90 mL of Buffered Peptone Water (BPW, Condalab, Madrid, Spain). Serial dilutions were prepared, and plate pouring was performed using De Man, Rogosa and Sharpe agar (MRS, Condalab, Madrid, Spain) and M17 agar (Himedia Laboratories, Mumbai, India). MRS and M17 were supplemented with 0.1% cycloheximide (AppliChem, Darmstadt, Germany), and they were incubated at 28 °C and 42 °C under anaerobic conditions using AnaeroGen™ Sachet (Thermo Fisher Scientific Oxoid, Basingstoke, UK). Colonies were selected from each medium, based on their distinct shape and color characteristics and then were further purified. The purified colonies were then stored at −80 °C with 20% (*v*/*v*) glycerol for future use.

### 2.3. Identification by MALDI-TOF MS

Isolated strains were subjected to MALDI-TOF MS analysis for identification. Stored strains were subcultured once using the respective medium each time. After incubation at the appropriate temperature for 24 h, the cultures were streaked on MRS agar (Condalab, Madrid, Spain) or M17 agar (Himedia Laboratories, Mumbai, India), depending on the specific medium in which each strain was initially isolated during the sampling process and incubated again for 24 h. Using a sterile toothpick, single colonies were carefully spotted on a stainless-steel target plate, and 1 μL matrix of saturated solution of α-HCCA (α-cyano-4-hydroxycinnamic acid in 50% acetonitrile and 2.5% trifluoroacetic acid) (Bruker Daltonics, Bremen, Germany) was added on top of the spots. The samples were left to dry at room temperature and then analyzed with the MALDI-TOF MS AutoflexIII (Bruker Daltonics, Bremen, Germany) using the default parameter settings within the MALDI Biotyper software ver. 3.1 (Bruker Daltonics, Bremen, Germany). To ensure reproducibility, each isolate was spotted two times on the plate. The identification of the isolates relied on log (score) values ranging from 0 to 3, which were acquired from a search within the reference database. Values of ≥2.0 signified identification at the species level, values of ≥1.7 represented identification at the genus level, and values < 1.7 were considered as unidentifiable. 

### 2.4. DNA Extraction from Sfela Cheese Samples

DNA extraction from Sfela cheese samples was carried out using the DNeasy PowerFood Microbial Kit (Qiagen, Hilden, Germany) according to the manufacturer’s protocol with slight modifications. To summarize the procedure, 0.5 g of Sfela cheese was collected in ten sterile Eppendorf tubes (1.5 mL), and 750 μL of trisodium citrate [2% (*m*/*v*), pH 7.4] was added to each tube. The suspensions were then centrifugated at 12,000× *g* for 10 min at 4 °C, and the resulting pellet was washed several times with the citrate solution. After each centrifugation step, any remaining fat was removed, and all the pellets from each sample were combined into one tube. Next, the final pellets of each Sfela sample were treated with 500 μL of lysozyme (25 mg mL^−1^ in Tris-EDTA buffer at pH 8.0), 20 μL of mutanolysin (5 U mL^−1^), 15μL of RNase (10 mg mL^−1^) and 5 μL of lyticase (1 U μL^−1^) (all reagents from Sigma-Aldrich ChemieGmbh, Munich, Germany). The samples were then incubated at 37 °C for 1.5 h while vortexing every 15 min. After centrifugation at 10,500× *g* for two minutes, DNA extraction from the pellet was carried out following the manufacturer’s instructions provided with the kit. The resulting eluted DNA was stored at −20 °C. Subsequently, DNA concentration and the ratios of 260/280 nm and 260/230 nm were determined using NanoDrop™ One/OneC Microvolume UV-Vis Spectrophotometer (Thermo Fisher Scientific, Cleveland, OH, USA). Furthermore, DNA integrity was assessed through agarose gel electrophoresis.

### 2.5. Sequencing and Bioinformatics Analysis

Library preparation, Illumina sequencing and quality control for 16S rDNA and shotgun metagenomics were conducted by Beijing Genomics Institute (BGI) and Novogene (Beijing, China). Data was imported into the CLC genomics workbench 23.0.5 (Qiagen, Hilden, Germany), and at first, chimera removal and operational taxonomic unit (OTU) clustering were performed with the default parameters, as previously described [35]. The 16S rDNA OTU tables were constructed using the Silva database version 138.1, with 99% identity. A closed OTU picking was selected to avoid the high number of unidentified taxa. Rarefaction of reads was performed using default settings. Alpha and beta diversities were calculated using the total number of OTUs and principal coordinate analysis (PCoA) based on Bray–Curtis distances, respectively. For shotgun metagenomics, the initial taxonomic profiling of samples was conducted by aligning the shotgun reads against a microbial genome database after creating a taxonomic profiling index in the CLC genomics workbench with default parameters. Subsequently, the shotgun reads were assembled using the de novo assembly function with a minimum length of 1Kbp for all the cheese samples with the default settings. Bin identification and taxonomic assignment was performed by the BusyBee web server [36,37]. For this analysis, the default parameters were chosen as well. *Cellulosimicrobium cellulans* reads were excluded from all the datasets during the analysis as their presence is due to the lyticase used for yeast lysis during DNA extraction [38,39]. The reference genomes in RefSeq of the most abundant species were employed to construct recruitment plots using the RecruitPlotEasy software with the default settings [40]. The initial step of mapping the sequence reads against the reference genomes was performed with the CLC genomics workbench. The specific reference genomes chosen for the analysis were those of *Str. thermophilus* CIRM 65, *L. lactis* LAC460, and *Tetragenococcus halophilus* MJ4. Finally, functional analysis of the assembled metagenomic reads was conducted using the CLC genomics workbench, for annotation and functional characterization of genetic sequences using the Gene Ontology (GO) molecular function terms. GO molecular function terms were visualized through heatmap construction using Euclidean distance for the first 25 molecular functions [41].

## 3. Results and Discussion

### 3.1. Identification of Isolates via MALDI-TOF MS Analysis

A total of 140 isolates from all the samples were selected based on their morphologies and growth at different temperatures. These isolates were subsequently identified using MALDI-TOF MS analysis, as shown in Table 1. The results revealed that the isolated species originated from various genera within LAB. The identification was considered successful, as all the species matches accounted for a score near or higher than 2.0. Among the total microbes analyzed from the four Sfela samples, the most frequent species were *E. faecium* (44.3%), *Lcb. plantarum* (23.6%), *Lvl. brevis* (10%), *P. pentosaceus* (8.6%), *Str. thermophilus* (5%), *Leu. mesenteroides* (3.6%) and *Lct. paracasei* (1.4%). Other bacteria were also identified, including *Enterococcus gallinarum*, *Latilactobacillus curvatus*, *Lacticaseibacillus rhamnosus*, *Streptococcus gallolyticus* and *Staphylococcus epidermidis*, but with lower abundances (<1%). Our results are in general accordance with previous studies conducted to identify LAB in white brined cheeses using MALDI-TOF MS analysis. More specifically, *E. faecium, Leu. mesenteroides* and *P. pentosaceus* have been isolated from white brined Serbian artisanal cheeses [42]. Furthermore, in the same study there were additional bacterial species that were absent from Sfela cheese, like *Macrococcus caseolyticus* and *E. faecalis* among others. In another study on the Spanish cheeses type “Torta”, researchers also identified some common bacteria shared with Sfela cheese, which included *Ltl. curvatus*, *Lct. paracasei*, *Lcb. plantarum*, *Lct. rhamnosus* and *Leu. mesenteroides* [43]. *Lcb. plantarum* is a rather frequent bacterial species of white brined cheeses that has been also identified with MALDI-TOF MS in Touloumotyri [44]. Finally, the findings most closely resembling ours were those of Feta cheese which was examined for its microbial shift during ripening [45]. During the initial ripening, some of the dominant lactobacilli species were *Lcb. plantarum*, *Lvl. brevis*, *Lct. rhamnosus*, *Lct. paracasei* and *Ltl. curvatus*, while the most frequently encountered *Enterococcus* species were *E. faecalis*, *E. faecium* and *E. durans*. *Str. thermophilus* and *P. pentosaceus* were also present in significant quantities during the first three months of ripening, while *Staph. epidermidis* was only detected after six months [45]. It is evident that common LAB species may be present in different white brined cheeses, but variations can also occur.

### 3.2. 16S rDNA Amplicon Sequencing

**Figure 1:**
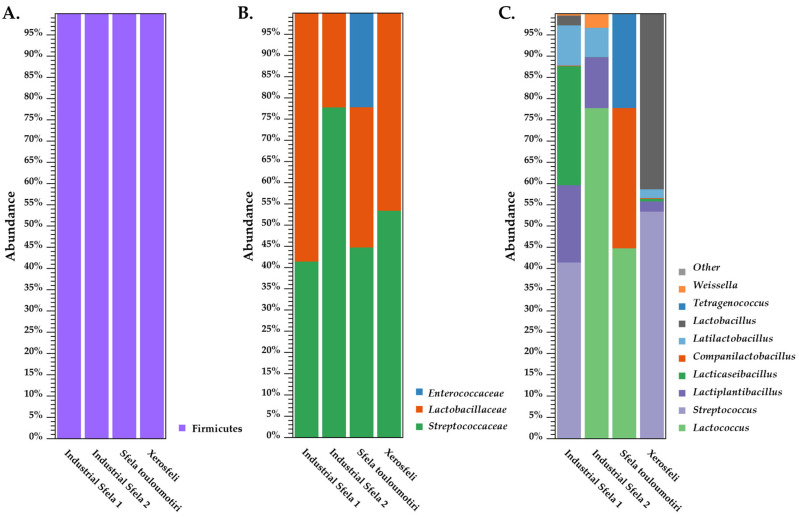
Illustration of the taxonomic distribution of industrial Sfela cheese and its artisanal variants, Sfela touloumotiri and Xerosfeli, using 16S rDNA amplicon sequence data. The samples were analyzed at the phylum (**A**), family (**B**), and genus (**C**) levels.

The next step of our analysis was the 16S rDNA amplicon sequencing of all four cheese samples. The clustering analysis of 16S rDNA reads, as shown in Figure 1A, reveals that the Firmicutes phylum was predominant across all samples, indicating that the Sfela cheese environment provides favorable conditions for the proliferation of Gram-positive bacteria. Some examples of Firmicutes commonly found in white brined cheeses include members of the *Lactobacillaceae* family and *Streptococcus* sp. The LAB are known for their role in the fermentation of dairy products and their contribution to the development of specific flavors, textures, and aromas [46]. Previous research conducted on Feta cheese has also reported the prevalence of the Firmicutes phylum in the bacterial microbiota [33]. In addition to Feta, Firmicutes was described to clearly dominate in various Greek PDO cheeses, including Anevato, Batzos, Galotiri, Kalathaki Limnou and Kopanisti [29].

The bacterial composition was more diverse at the family level, as shown in Figure 1B. More specifically, *Streptococcaceae* emerged as the dominant family in industrial Sfela 2 and Sfela touloumotiri, accounting for 79% and 43.7%, respectively. In industrial Sfela 1 and Xerosfeli, it also appeared with high percentages, particularly 39% and 48%, but in both samples, it was the second most prevalent family. The family that predominated in industrial Sfela 1 (61%) and Xerosfeli (49.9%) was *Lactobacillaceae*, which was the second highest population in industrial Sfela 2 (21%) and in Sfela touloumotiri (30%). Sfela touloumotiri contained two additional families, namely *Enterococcaceae* and *Staphylococcaceae*, with abundances of 23.3% and 3%, respectively. *Lachnospiraceae* is a family that only appeared in Xerosfeli but with a low abundance of 2%. 

The differences between the samples became even more noticeable when compared for the most dominant genera (Figure 1C). In the industrial Sfela 1 and the Xerosfeli samples, the primary member of the *Streptococcaceae* family was the genus *Streptococcus* with 43% and 53% abundances, respectively. In contrast, the most abundant genus of the same family in the other two samples was *Lactococcus* representing 78% of the industrial Sfela 2 and 45% of Sfela touloumotiri. In these two samples, *Streptococcus* was present in low percentages (<1% in industrial Sfela 2 and 3.9% in Sfela touloumotiri), as was also observed in the case of *Lactococcus* detected in industrial Sfela 1 and Xerosfeli (<1%). *Lactiplantibacillus* was present in industrial Sfela 1, industrial Sfela 2 and Xerosfeli with 18.9%, 10.9% and 2% abundance, respectively. *Lacticaseibacillus* was identified only in industrial Sfela 1, occupying a notable percentage of 28.2% of the total sample. The genus *Companilactobacillus* appeared only in Sfela touloumotiri with a relatively high abundance (27.9%), while in the rest of the samples, its presence was not significant (<1%). On the contrary, *Latilactobacillus* appeared in industrial Sfela 1, industrial Sfela 2 and Xerosfeli with varying abundances of 2–9%, while in Sfela touloumotiri with <1%. *Lactobacillus* is another genus of the same family, which was detected in industrial Sfela 1, accounting for 2.4% and in Xerosfeli 36.9%. Finally, *Weissella* was identified in industrial Sfela 2, Sfela touloumotiri and Xerosfeli with 3.1%, 2.1% and 3.5%, respectively. 

Regarding the most prevalent bacterial genera reported in Sfela samples, the results are in accordance with studies employing amplicon sequencing on various types of white brined cheeses. More specifically, *Lactococcus*, *Streptococcus* and *Lactobacillus* were consistently identified as the significant genera in Tulum, Minas Gerais, Feta, Halloumi and Halitzia cheeses [25,30,31,32,34,47]. Additionally to these three frequent cheese genera, *Lactiplantibacillus* and *Lacticaseibacillus* were also identified with 16S rDNA amplicon sequencing in Feta [35] and *Weissella* in Minas Gerais and Tulum cheeses [25,30]. Nevertheless, the distribution of these bacteria varied between the cheeses, and there were also differences concerning the presence of less abundant genera, including *Bifidobacterium*, *Pediococcus* and *Pseudomonas* which uniquely appeared in specific studies [25,30,34,35]. As already mentioned, white brined cheeses usually contain NSLAB that originate from raw milk or contamination occurring after heat-treatment. The mesophilic lactobacilli are the predominant type of NSLAB found in white brined cheeses [12,48], which could explain the high frequency of these genera in Sfela samples. Interestingly, the genus *Tetragenococcus* of the *Enterococcaceae* family was exclusively found in Sfela touloumotiri, showing a significant abundance of 23.3%. This genus consists of halophilic LAB, thriving in high-salt environments, which makes them well-suited for white brined cheese environments, and they have also been identified in cheese brines [49,50]. Some species show proteolytic and lipolytic activities, contributing to the development of sensory characteristics in ripened cheese products [51]. In a previous study, *T. halophilus* has been suggested as a potential starter culture for fermented foods with high salinity [52].

**Figure 2:**
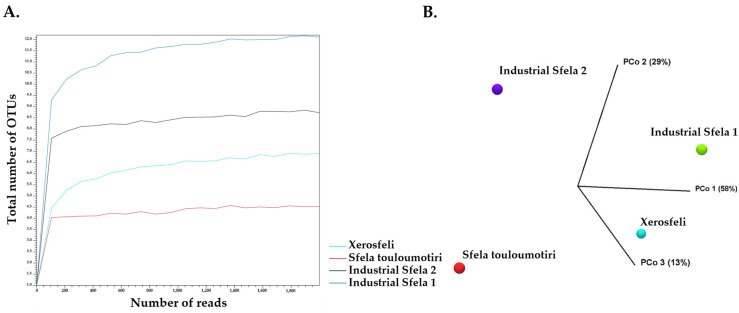
Alpha diversity analysis of 16S rDNA reads for a maximum depth of 18,000 read counts based on the total number of OTUs at genus level (**A**) and beta diversity presented in a Principal Coordinate Analysis (PCoA) employing the Bray—Curtis distances for the samples (**B**).

The analysis at the phylum level did not reveal any taxonomic differences, but the family and genus levels provided a deeper insight into the bacterial distribution among the samples. The alpha diversity analysis (Figure 2A) confirmed that the sequence depth was adequate for each sample. Furthermore, beta diversity revealed that some samples could be clustered together when assessing the variation at the genus level. More specifically, industrial Sfela 1 and Xerosfeli were classified together and separately from industrial Sfela 2 and Sfela touloumotiri according to PCo1 (Figure 2B). 

### 3.3. Identification of Sfela Microbiome at Species Level Using Shotgun Metagenomics Analysis

**Figure 3:**
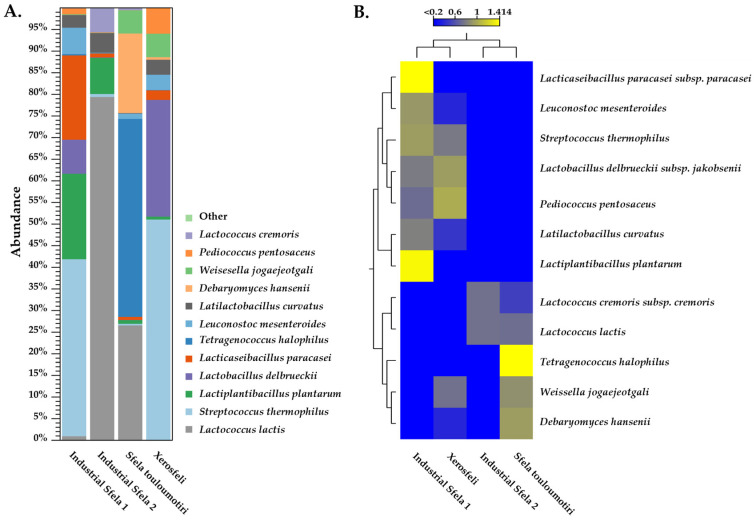
Illustration of the taxonomic distribution of industrial Sfela cheese and its artisanal variants, Sfela touloumotiri and Xerosfeli, using shotgun metagenomics sequence data. The samples were analyzed at the species level (**A**). Heat-map analysis of the species identified in industrial Sfela 1, industrial Sfela 2, Sfela touloumotiri and Xerosfeli (**B**).

Species-level identification of the Sfela cheese microbiome was achieved by shotgun metagenomics. We compared the reads from these samples against a representative database of bacterial and fungal genomes (Figure 3). The species-level analysis revealed that industrial Sfela 1 was mainly characterized by the presence of *Str. thermophilus* (40.74% abundance), which appeared in even higher abundance in Xerosfeli at 50.5% in particular. 

*L. lactis* predominated in industrial Sfela 2, reaching up to 79.2%, and it also appeared in Sfela touloumotiri with 27.1% abundance. *T. halophilus*, the most frequent species in Sfela touloumotiri, occupied 45.9% of the total sample, while it could not be detected in the rest of the samples (<1% abundance). *Lcb. plantarum* was another species that exhibited significant variations among the samples, and in particular, it reached 19.7% in industrial Sfela 1 and 8.3% in industrial Sfela 2, while it appeared in less than 1% in the two artisanal cheeses. Industrial Sfela 1 and Xerosfeli shared two additional species, namely *Lb. delbrueckii* reaching 8.1% and 26.6% and *Lct. paracasei* with 19.7% and 2.4% abundance in industrial Sfela 1 and Xerosfeli, respectively. *Ltl. curvatus*, was found in notable amounts in industrial Sfela 1 (2.9%), industrial Sfela 2 (4.5%) and Xerosfeli (3.3%). *Leu. mesenteroides* was also detected in substantial abundancies of 6.4%, 1.2% and 3.3% in industrial Sfela 1, Sfela touloumotiri and Xerosfeli, respectively. Several other bacterial species were detected in varying abundances, including *P. pentosaceus*, *Lactococcus cremoris* and *Weissella jogaejeotgali*. Of note, some *Bifidobacterium* spp. were detected but their abundance was low (<1%). *D. hansenii* was the only yeast species found in abundance exceeding 1%. Notably, it appeared in a high percentage of 13.5% in Sfela touloumotiri. It was also present in the remaining samples, occupying < 1% of the microbial population. Additional yeast species found in the samples, but with low abundances (<1%), included *Neurospora crassa*, *Tor. delbrueckii* and *Debaryomyces nepalensis*.

As previously described, the diverse array of bacterial species found across samples verifies the rich and dynamic microbial profile of white brined cheeses like Sfela. *Str. thermophilus* has been previously identified in various cheese types, particularly in those produced through fermentation processes involving high temperatures. Its prevalence and significance are noteworthy in Feta cheese [35,45] and other Mediterranean cheeses, like Italian Grana Padano [53] and Parmigiano-Reggiano [54]. Its high abundance can be attributed to the potential addition of starter cultures. The dominance of *L. lactis* in the industrial Sfela 2 is in agreement with its widespread occurrence in various white brined cheeses, including Feta cheese [35,55]. 16S rDNA amplicon sequencing and shotgun metagenomics consistently identify *L. lactis*, *Str. thermophilus*, and *Lb. delbrueckii* as the three most prevailing species in white brined cheeses [24,26,27]. *Leu. mesenteroides* has been previously identified in the traditional Montenegrin brine cheese [56] and in Feta cheese through shotgun metagenomics analysis [35]. *W. jogaejeotgali*, initially found in traditionally fermented Korean jogae jeotgal [57], exhibits resistance to osmotic stress and tolerance to acidic conditions. Recent discoveries have placed *W. jogaejeotgali* as part of the cheese microbiome [26,58], though other species of this genus have been also isolated from dairy environments in previous studies [51,59]. Furthermore, our findings align with previous studies on Feta, Domiati, and Halloumi cheeses, indicating a consistent presence of non-starter lactobacilli in mature samples [12,60,61].

The results from the shotgun analysis are generally in accordance with the 16S rDNA amplicon sequencing analysis, which provided insights into the bacterial composition of the samples up to the genus level. Moreover, the shotgun analysis at the species level is partially consistent with the culture-based analysis and the identification of the isolates with MALDI-TOF MS after culture-based isolation. In particular, both approaches revealed the presence of *Str. thermophilus*, *Lcb. plantarum*, *Lct. paracasei* and *Leu. mesenteroides*. However, it is worth noting that the data regarding the species with abundances exceeding 1% did not always match the culture-based analysis results. For instance, enterococci were found in high populations among the identified isolates, in contrast to the metagenomics results in which they were <1% abundant. Shotgun analysis revealed the presence of highly abundant species that could not be isolated and characterized by the cultured-based analysis. This discrepancy may be attributed to the selection of colonies based on morphological variations rather than a randomized selection. Consequently, the identified taxa through culture-based analysis may appear with different frequencies than those found through metagenomics analysis. Another aspect to consider is that both amplicon and shotgun sequencing techniques can detect bacteria even if they are no longer viable or able to be cultivated, as previously suggested [62]. Furthermore, during our study, cell culture conditions might have provided an environment favoring the growth and subsequent selection of certain species from the microbiome present in the Sfela samples. In addition, the inability to isolate some taxa with high abundances, as indicated by the shotgun metagenomics and the 16S amplicon sequencing, may suggest that some of them could have been in a state where they are “viable but not culturable” (VBNC). For instance, in one study, strains of *L. lactis* used in cheese making were found to be viable with RT-qPCR, but they could not be grown on M17 throughout the ripening period [63]. Another study supporting the VBNC condition of *L. lactis* in Lebanese fermented milk products demonstrated that despite the inability to isolate the species on M17, the addition of goat milk in the culture medium facilitated its recovery [64]. This phenomenon could potentially be attributed to the depletion of carbohydrates, possibly occurring during ripening or storage, leading to *L. lactis* entering a VBNC state [65]. 

Comparing the most dominant species within the four samples revealed that the bacterial diversity in industrial Sfela 1 and Xerosfeli exhibited similarity and industrial Sfela 2 was grouped together with Sfela touloumotiri, as illustrated in Figure 3B. Among the bacterial species, the presence of *Str. thermophilus*, *Lb. delbrueckii* and *P. pentosaceus* were responsible for grouping industrial Sfela 1 and Xerosfeli. The grouping of industrial Sfela 2 and Sfela touloumotiri relied on the presence of two distinct lactococci, i.e., *L. lactis* and *L. cremoris*. In addition, *T. halophilus* was the characteristic species in Sfela touloumotiri. Furthermore, *Lcb. plantarum*, *Lactiplantibacillus paraplantarum*, and *Lct. paracasei* were prevalent in industrial Sfela 1. 

**Figure 4:**
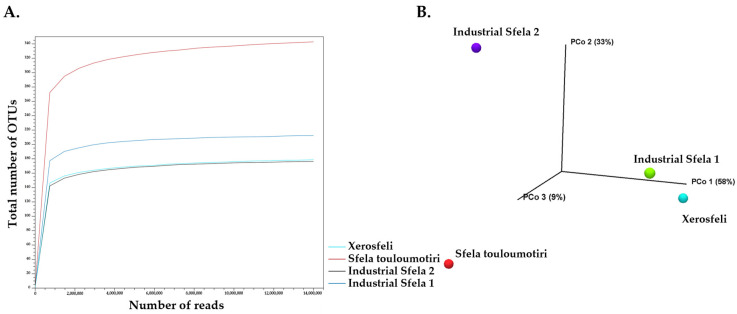
Alpha diversity analysis of shotgun reads for a maximum depth of 14,000,000 read counts based on the total number of OTUs (**A**) and beta diversity presented in PCoA employing the Bray—Curtis distances for the samples (**B**).

Alpha diversity was also calculated for the shotgun metagenomic reads. As indicated in Figure 4A, the depth of the sequencing was again adequate for each sample. As shown in Figure 4B, the beta diversity analysis of the shotgun metagenomics sequencing segregated the samples in a manner similar to the heatmap analysis of the most dominant species. More precisely, industrial Sfela 1 and Xerosfeli were grouped together and distinct from industrial Sfela 2 and Sfela touloumotiri according to PCo1.

### 3.4. Binning of Metagenome-Assembled Contigs of Sfela Cheese Samples—Construction of Recruitment Plots

**Figure 5:**
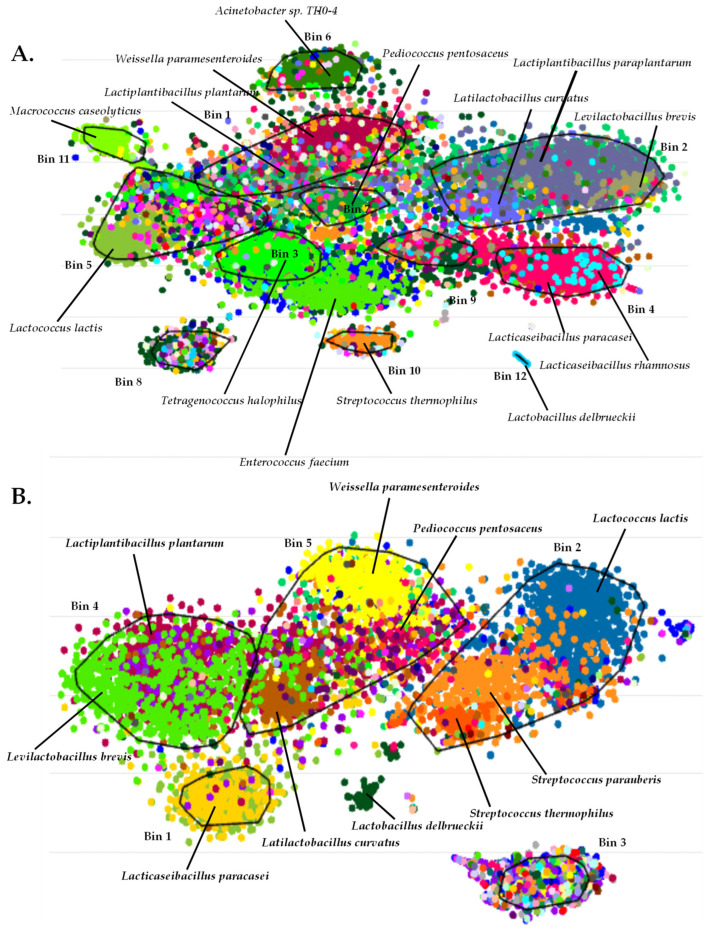

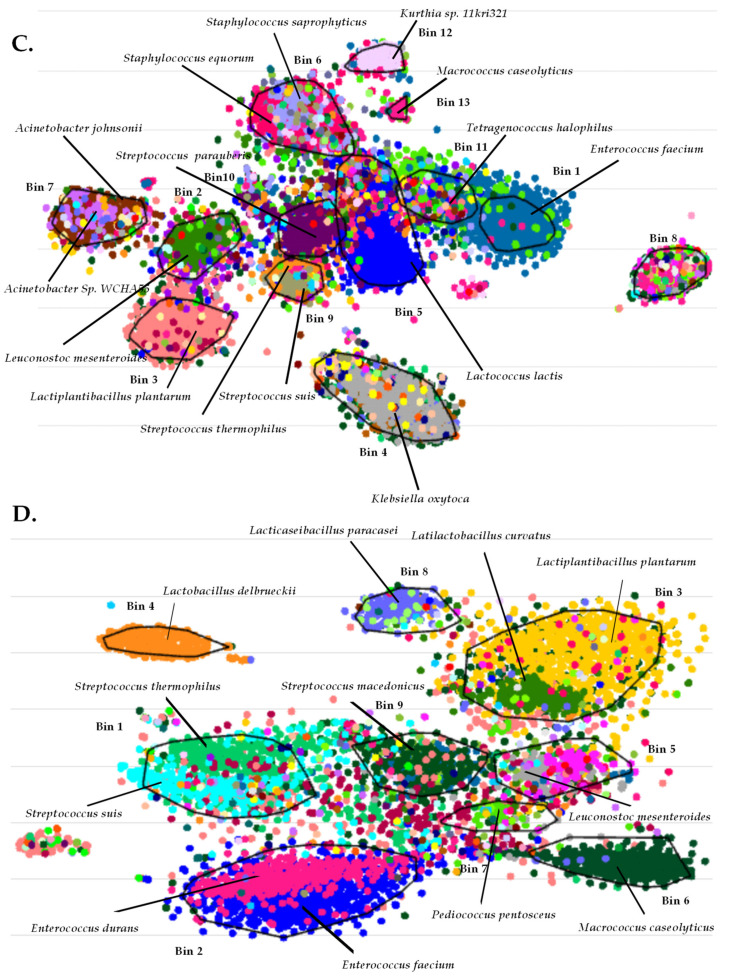
Bins of metagenomics scaffolds of industrial Sfela 1 (**A**), industrial Sfela 2 (**B**), Sfela touloumotiri (**C**) and Xerosfeli (**D**). The figure indicates the identified species’ contigs distributed in different bins represented by dots of the same color.

To identify metagenome-assembled genomes (MAGs), we performed binning using the assembled contigs of each sample (Figure 5). As shown in Table 2, we were able to get several bins with high completeness. Still, in most cases, these were accompanied by high levels of contamination and/or strain heterogeneity. However, there were some exceptions of high-quality bins, like Bin 12 in industrial Sfela 1, as well as Bin 4, Bin 6 and Bin 8 in Xerosfeli. These bins were identified as *Lb. delbrueckii* (Bin 12 and Bin 4), *M. caseolyticus* (Bin 6) and *Lct. paracasei* (Bin 8). 

The taxonomic evaluation of all the bins and contigs agreed with our species identification findings by the shotgun metagenomics analysis of single reads presented above. However, BusyBee analysis demonstrated the presence of bins of contigs corresponding to species appearing with less than 1% abundance in the shotgun analysis. This was the case for the species *Lcb. paraplantarum*, *E. durans*, *Streptococcus parauberis*, *M. caseolyticus* and other less frequent species found dispersed among the bins of the different samples. *Staphylococcus equorum* was one of the aforementioned species, which is a non-pathogenic staphylococcal member and was found to contribute to the intense flavor profiles found in cheeses [66] and has been suggested as a starter culture for the manufacturing of semi-hard cheeses [67]. The binning process also revealed the presence of some additional bacteria, including *Acinetobacter johnsonii* and *Acinetobacter* sp. (TTH0-4, WCHA55), which have been related to cheese spoilage [68] and can act as opportunistic pathogens [69]. Despite the fact that *Str. parauberis* has been previously reported in cheese environments [33,70] and sheep milk [71], its presence may be unwanted as it has been connected with cases of bovine mastitis [72]. *Streptococcus suis* is another bacterium that has been identified in cheese samples [73], but its occurrence may pose safety concerns as a zoonotic pathogen [74]. Detecting these bacteria in Sfela samples can raise concerns regarding the hygienic conditions during the cheese production and it underlines the importance of an improved surveillance and monitoring of the process. 

**Figure 6:**
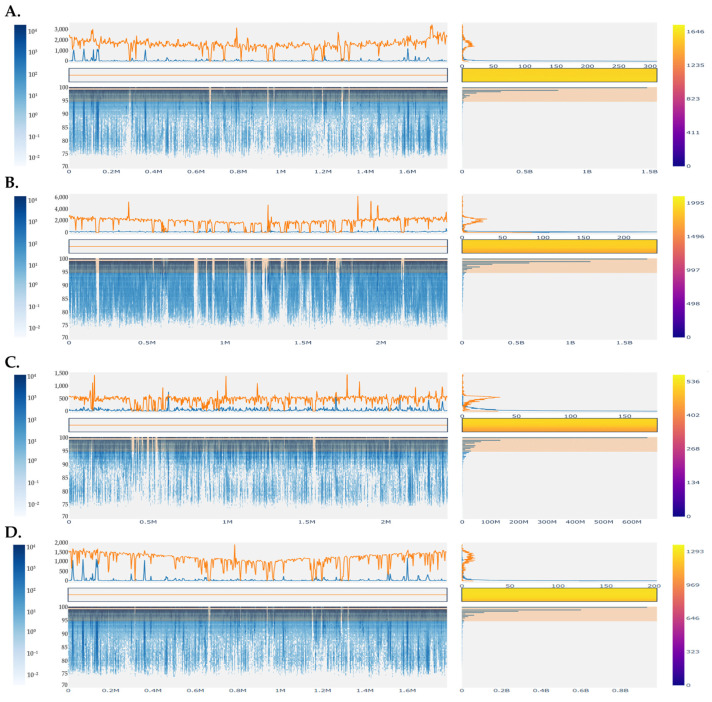
Recruitment plots of metagenomic reads from industrial Sfela 1 against the sequence of the reference genome of *Str. thermophilus* (CIRM 65) (**A**), industrial Sfela 2 against the genomic sequence of the reference strain of *L. lactis* (LAC460) (**B**), Sfela touloumotiri against the genomic sequence of the reference strain of *T. halophilus* (MJ4) (**C**), and Xerosfeli against the genomic sequence of the reference strain of *Str. thermophilus* (CIRM 65) (**D**). Bottom left panel: reads that have been aligned to the genome. The *X*-axis represents the position in the genome, while the *Y*-axis represents the percent of identity. The color of the data points corresponds to the number of reads that match a particular position on the genome with a specific level of identity indicated from the left bar. Upper left panel: sequencing depth across different regions of the genome. Bottom right panel: number of nucleotide bases at different levels of nucleotide identity. The *Y*-axis represents the nucleotide identity, and the *X*-axis represents the number of bases. Upper right panel: histogram of the sequencing depth. Orange and blue lines represent matches with nucleotide identity above and below the 95% cutoff, respectively.

To surpass the problem of low-quality bins within our dataset and more specifically, to determine the MAGs of the most prevalent species in each sample, we constructed recruitment plots of sequencing reads against the reference genomes of *Str. thermophilus* (for industrial Sfela 1 and Xerosfeli), *L. lactis* (for industrial Sfela 2) and *T. halophilus* (for Sfela touloumotiri). As shown in Figure 6, this approach enabled the assembly of nearly complete draft genomes for each of the reference genomes. Interestingly, a significant number of reads exhibited alignments with an identity exceeding 95%, while also displaying a high average depth of sequencing coverage.

### 3.5. Functional Analysis of Metagenome Contigs

**Figure 7:**
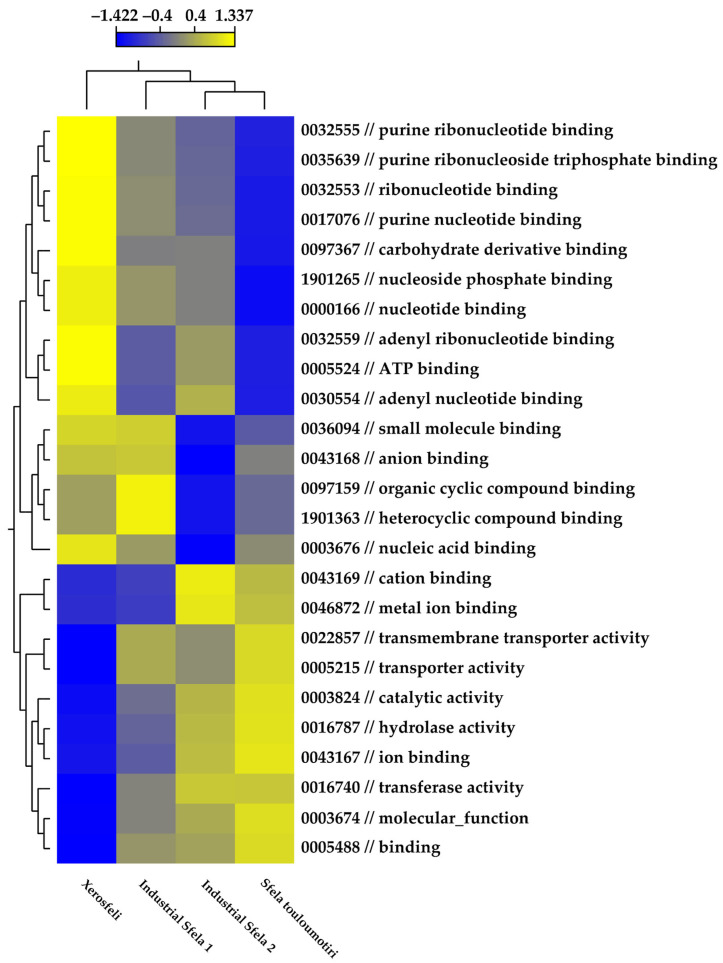
Heat map representing the results of functional analysis conducted on Sfela cheese samples according to GO molecular function. The analysis was conducted using the Euclidean clustering distance to group functional properties and the different cheese samples.

Functional analysis of the assembled shotgun metagenomes was performed in the CLC genomics workbench. Heatmap analysis demonstrated that different functions had different distributions in each sample (Figure 7). The grouping between industrial Sfela 2 and Sfela touloumotiri was again evident. In contrast, industrial Sfela 1 and Xerosfeli did not form a distinct group. It should be mentioned that our analysis concerns the presence/absence of functions rather than their actual activity during the production of these cheese samples, given that they derive from a metagenomics rather than a metatranscriptomics dataset. Further research is required to explore the roles of the identified activities and to develop a more comprehensive image of the molecular functions contributing to the production of the different Sfela cheese types.

## 4. Conclusions and Future Perspectives

Sfela cheese has a PDO status, signifying that its distinct organoleptic characteristics are firmly connected to the Messinia and Laconia regions in the Peloponnese. In our study, we utilized a combination of 16S rDNA amplicon sequencing and shotgun metagenomics to evaluate four different Sfela samples: two produced on an industrial scale under PDO regulation and two non-PDO variants of Sfela cheese. Notably, industrial Sfela 1 and Xerosfeli were dominated by *Str. thermophilus* and *Lb. delbrueckii* subsp. *bulgaricus*, whereas industrial Sfela 2 and Sfela touloumotiri were characterized both by high populations of *L. lactis*, while *T. halophilus* prevailed in Sfela touloumotiri. As already mentioned, bacteria employed as starter cultures may persist in the first stages of cheese fermentation, while NSLAB may prevail at the later ripening stages due to the low pH and the elevated salt content. The dominance of *Str. thermophilus* and *Lb. delbrueckii* subsp. *bulgaricus* in industrial Sfela 1 and Xerosfeli may be attributed to production under strict hygiene conditions, enabling these bacteria to thrive during ripening against other microbes [35]. Metagenomics analysis also revealed that NSLAB were detected in all samples, but they were not among the most abundant species. The presence of yeast was rather scarce, as indicated by shotgun metagenomics. The binning of scaffolds did provide several sequence bins of varying completeness and quality. Notably, this analysis unveiled the presence of *Acinetobacter* spp., *Str. parauberis* and *Str. suis* in Sfela. This suggests the need for stricter surveillance during production, ensuring adherence to safety protocols, and compliance with PDO regulations to ensure food safety and consumers’ health. Standardizing the production of traditional cheese can also potentially enhance their demand [75,76]. Omics approaches are critical as they can provide a very detailed picture of the microorganisms participating throughout the whole procedure of cheese production. Culture-dependent techniques also provide essential information about the product’s dominant microbiome, and thus the combination of both methods can shed light on the dynamic changes that the microbial composition undergoes along with the functional pathways they carry. The information presented here provides a first framework concerning the intricate microbial communities that may exist in Sfela cheese. Further exploration may be needed to unveil a more comprehensive map of the Sfela cheese microbiome and its impact on sensory attributes. The construction of a pool of naturally occurring strains with desirable properties may lead to the isolation of novel strains for use as valuable starters or adjuncts specific to the production of Sfela cheese. Such research will not only expand our understanding of the Sfela cheese microbiome but also holds potential for enhancing its quality and safety in the future. Our study may also encourage the application of shotgun metagenomics to study the microbiomes of other types of white brined cheese produced in different parts of the world.

## Figures and Tables

**Table 1:** Species identification via MALDI-TOF MS.

Identified Species via MALDI-TOF MS	Total Number
*Enterococcus faecium*	62/140 (44.3%)
*Lactiplantibacillus plantarum*	33/140 (23.6%)
*Levilactobacillus brevis*	14/140 (10%)
*Pediococcus pentosaceus*	12/140 (8.6%)
*Streptococcus thermophilus*	7/140 (5%)
*Leuconostoc mesenteroides*	5/140 (3.6%)
*Lacticaseibacillus paracasei*	2/140 (1.4%)
*Enterococcus gallinarum*	1/140 (0.7%)
*Latilactobacillus curvatus*	1/140 (0.7%)
*Lacticaseibacillus rhamnosus*	1/140 (0.7%)
*Streptococcus gallolyticus*	1/140 (0.7%)
*Staphylococcus epidermidis*	1/140 (0.7%)

**Table 2:** Quality of bins obtained from the assembled metagenomes of Sfela cheese samples.

Sample	Bin	Completeness *	Contamination *	Strain Heterogeneity *
Industrial Sfela 1	1	93.69	127.03	7.69
	2	95.50	225.23	9.83
	3	80.18	36.94	8.77
	4	82.88	9.91	60.00
	5	87.39	95.50	2.67
	6	81.08	17.12	13.04
	7	72.97	9.91	7.69
	8	29.73	0.90	0.00
	9	8.11	0.00	0.00
	10	45.95	0.00	0.00
	11	91.89	63.06	7.80
	12	91.89	0.00	0.00
Industrial Sfela 2	1	20.72	1.80	50.00
	2	79.28	122.52	14.80
	3	11.71	3.60	100.00
	4	93.69	80.18	12.73
	5	94.59	161.26	10.29
Sfela touloumotiri	1	48.65	11.71	40.00
	2	94.59	207.21	6.35
	3	57.66	14.41	18.75
	4	82.88	8.11	33.33
	5	67.57	25.23	28.57
	6	91.89	81.08	62.04
	7	86.49	12.61	50.00
	8	93.69	74.77	6.52
	9	72.07	18.02	40.00
	10	54.95	5.41	44.44
	11	91.89	46.85	8.33
	12	95.50	36.94	12.24
	13	72.97	0.90	0.00
Xerosfeli	1	54.95	29.73	26.32
	2	94.59	87.39	63.79
	3	95.50	85.59	10.17
	4	90.09	0.00	0.00
	5	95.50	138.74	3.70
	6	93.69	9.01	25.00
	7	68.47	2.70	0.00
	8	95.50	3.60	0.00
	9	61.26	18.02	39.13

* determined through analysis with the BusyBee web tool.

## Data Availability

Publicly available datasets were analyzed in this study. This data can be found here: [SRA under BioProject IDs: PRJNA1072025, PRJNA1072084].
